# Growth control of *Marchantia polymorpha* gemmae using nonthermal plasma irradiation

**DOI:** 10.1038/s41598-024-53104-1

**Published:** 2024-02-07

**Authors:** Shoko Tsuboyama, Takamasa Okumura, Pankaj Attri, Kazunori Koga, Masaharu Shiratani, Kazuyuki Kuchitsu

**Affiliations:** 1 Department of Applied Biological Science, Tokyo University of Science, 2641 Yamazaki, Noda, Chiba 278-8510 Japan; 2 Faculty of Information Science and Electrical Engineering, Kyushu University, 744 Motoka, Fukuoka City, Fukuoka 819-0395 Japan; 3 Center of Plasma Nano-Interface Engineering, Kyushu University, Fukuoka, 819-0395 Japan

**Keywords:** Electrical and electronic engineering, Plasma physics

## Abstract

Several studies have documented that treatment by cold atmospheric pressure plasma (CAPP) on plants foster seed germination and growth in recent years. However, the molecular processes that underlie the action of CAPP on the seeds and plants remain mostly enigmatic. We here introduce gemmae of *Marchantia polymorpha,* a basal liverwort, as a novel model plant material suitable for CAPP research*.* Treating the gemmae with CAPP for a constant time interval at low power resulted in consistent growth enhancement, while growth inhibition at higher power in a dose-dependent manner. These results distinctly demonstrate that CAPP irradiation can positively and negatively regulate plant growth depending on the plasma intensity of irradiation, offering a suitable experimental system for understanding the molecular mechanisms underlying the action of CAPP in plants.

## Introduction

Cold atmospheric pressure plasma (CAPP) has gained significant attention in agricultural applications because it has been reported to enhance seed germination, sterilize seeds and their products, prevent plant diseases, and promote plant growth^1–7^. On the other hand, other reports using seedlings and shoots of *Arabidopsis thaliana* provide evidence that CAPP irradiation does not show a significant or negative effect on plant growth^8,9^. To optimize the use of CAPP technology in agriculture, it is essential to understand the molecular events induced in plant cells during and after CAPP irradiation as well as the molecular mechanisms underlying these effects. CAPP generation in the atmosphere containing oxygen, nitrogen, and water molecules produces various reactive oxygen and nitrogen species (RONS)^4^.

Reactive oxygen species (ROS) are generated during aerobic respiration and photosynthesis, particularly under stress conditions in intracellular organelles such as chloroplasts, mitochondria and peroxisomes. These ROS can attack biomolecules such as proteins, nucleic acids and lipids, making them highly toxic and capable of causing growth inhibition and cell death. Nevertheless, not only reactive nitrogen species (RNS) such as nitric oxide (NO) but also ROS are deliberately produced in a highly regulated manner to act as critical signaling messengers that play vital roles in controlling a broad range of physiological processes, such as cellular growth and development, as well as adaptation to environmental changes^6^. For example, the plasma membrane NADPH oxidases (Nox)/respiratory burst oxidase homologs (Rboh) produce superoxide anion radical (O_2_^−^) into the cell wall^6^, which is then converted to hydrogen peroxide (H_2_O_2_) and hydroxyl radicals (^·^OH)^7^. Based on these findings, it has become clear that CAPP-generated RONS have toxic effects while, at the same time, could also act as signaling molecules in plants. However, the detailed mechanism underlying the CAPP effect remained mostly unknown.

To date, our research group^10–13^ and other leading groups^14–17^ of the world have reported improvements in the germination and seedling growth of a variety of plant species, including *Raphanus sativus*, *Arabidopsis thaliana,* wheat, sunflower, soybean, barley, and sunflower*.* However, it should be noted that the effects of CAPP irradiation on seeds are highly dependent on various factors such as seed color^13^ and the physiological state of the seeds including water content of seeds^18^. Furthermore, levels of phytohormones and dormancy can greatly fluctuate depending on the growth environment of parent plants, storage conditions, and storage period. These variabilities of plant samples prevent consistent experimental results to elucidate the molecular mechanisms underlying the effects of CAPP on plants.

We here introduce a dioecious liverwort (bryophyte), *Marchantia polymorpha*, as a novel experimental model plant that is highly suitable for CAPP research. This plant can be easily and stably supplied, and its physiological conditions can be strictly regulated in experimental laboratories, allowing for consistent results to be obtained.

*M. polymorpha* is a basal land plant^19–22^ and has recently gained attention as a new model plant among plant molecular biologists. Its whole-genome sequence, completed in 2017^23^, revealed that most genes are highly conserved in land plants, with much lower genetic redundancy compared to other model plants^21^ (*cf.* a comprehensive database MarpolBase, https://marchantia.info). Furthermore, many techniques for molecular experiments, such as transformation and genome editing methods, have been established^24^. Therefore, once a good experimental system to test the effect of CAPP is established, many molecular biological tools could be easily applicable to elucidate the molecular mechanisms for its action.

**Figure 1:**
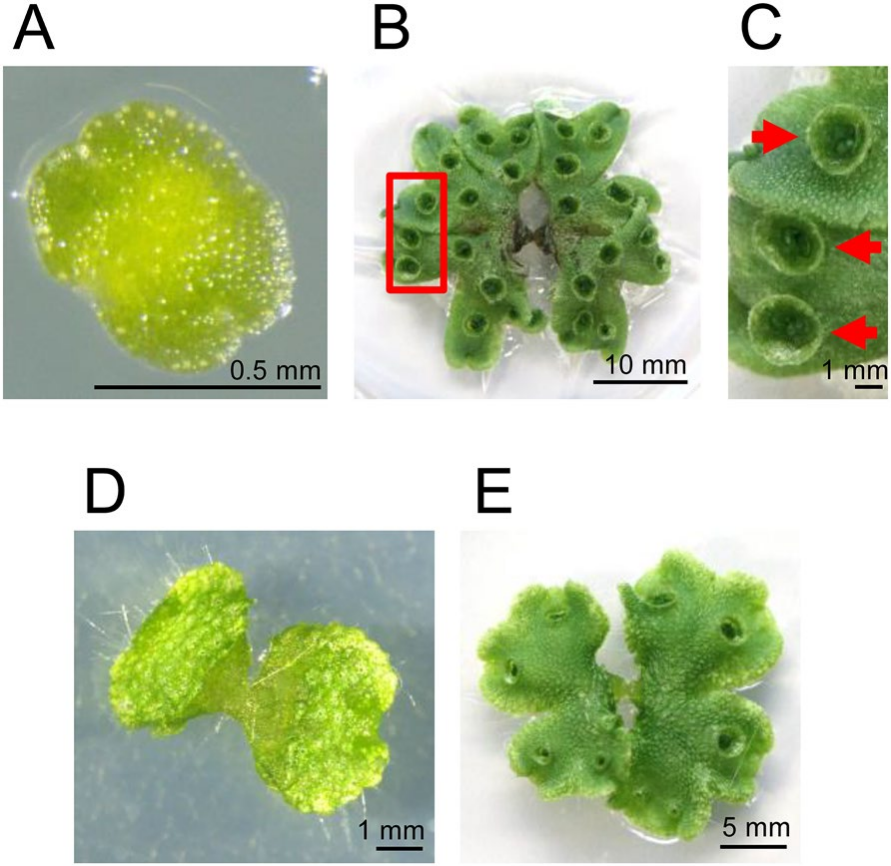
The pictures of tissue of *Marchantia polymorpha*. (**A**,**D**) The pictures were taken by a S8AP0 microscope (Leica Microsystems, Wetzlar, Germany) and a WRAYCAM-NOA2000 digital camera (WRAYMER, Osaka, Japan). (**B**,**C**,**E**) Pictures were taken by digital camera EOS M5 (Canon, Tokyo, Japan). (**A**) The gemma before culture. (**B**) The 3 weeks cultured gemmaling (matured thallus) on 1/2 Gamborg’s B5 supplemented with 1% sucrose, pH 5.5 (1% agar). (**C**) Enlarged picture inside the red square of B. Red arrows indicate gemma cups. Gemmae are developed in the gemma cup. (**D**) The 7 days cultured gemmaling on 1/2 Gamborg’s B5 supplemented with 1% sucrose, pH 5.5 (1% agar). (**E**) The 14 days cultured gemmaling on 1/2 Gamborg’s B5 supplemented with 1% sucrose, pH 5.5 (1% agar).

To the best of our knowledge, this is the first report to use the gemmae of *M. polymorpha* as a model plant material for CAPP irradiation. Gemmae are a convenient tissue that can be easily obtained, enable a unified genetic background (Fig. 1A)^25–27^. Gemmae are the clone of matured thalli obtained by asexual reproduction and develop from single cells in the gemma cup on a matured thalli (Fig. 1B, C)^28^. The gemmae grow to form an almost symmetrical shape (Fig. 1D), and finally, become thalli (Fig. 1E)^29^. Leveraging the advantages of *M. polymorpha* is expected to promote future mechanistic elucidation of plasma effects on plants. To utilize *M. polymorpha* to plasma science, it is imperative to establish a high-reproducible experimental system for controlling the growth of *M. polymorpha*.

In the present study, we have established an experimental system to irradiate gemmae of *M. polymorpha* with CAPP and showed that CAPP treatment for a constant time interval at low power resulted in consistent growth enhancement, whereas growth inhibition at higher power in a dose-dependent manner. Results suggest that CAPP irradiation could control plant growth positively and negatively depending on the plasma intensity of irradiation and provides a suitable experimental system to understand the molecular mechanisms for CAPP action in plants.

## Results

### Treatment condition of gemmae of *M. polymorpha* by SDBD

**Figure 2:**
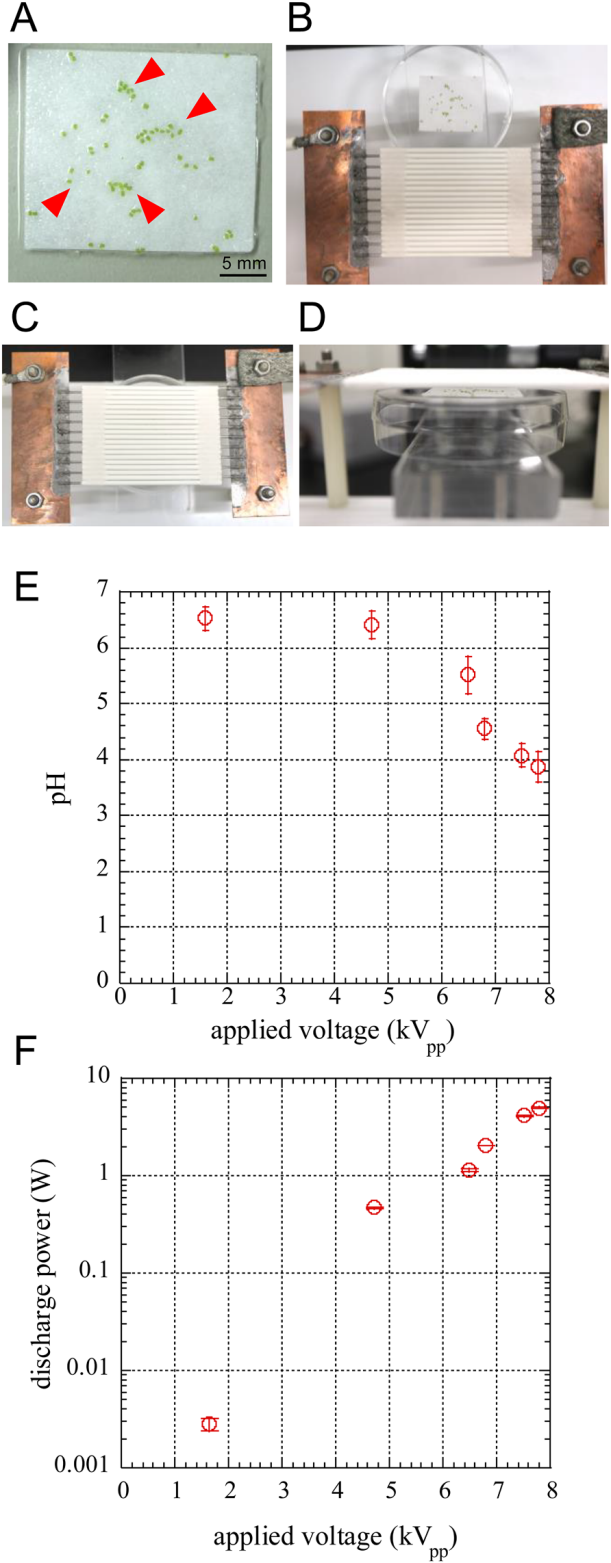
The pictures of gemmae of *Marchantia polymorpha* set under the SDBD device. (**A**–**D**) Pictures were taken by digital camera EOS M5 (Canon, Tokyo, Japan). (**A**) The gemmae on wet filter paper (approximately 2.5 mm × 2.5 mm) for SDBD plasma irradiation. Red arrow heads indicate gemmae. (**B**) The gemmae before set under SDBD device. (**C**) Top view of the gemmae set under SDBD device. (**D**) Side view of the gemmae set under SDBD device. (**E**) pH of SDBD plasma irradiated Milli-Q water in filter paper. pH was measured by LAQUAtwin-pH-22B (HORIBA Advanced Techno, Co., Ltd., Kyoto, Japan). (**F**) Discharge power consumed by SDBD plasma with the applied voltage. Discharge power consumed by SDBD plasma was measured by V-Q Lissajous method using a high voltage probe P6015A (Tektronix, Oregon, USA), a digital oscilloscope DS-5110B (Iwatsu, Tokyo Japan), and a capacitor of 100 nF inserted between the SDBD electrode and the ground.

For scalable dielectric barrier discharge (SDBD) plasma irradiation, several dozens of gemmae of *M. polymorpha* were put on a wet filter paper (approximately 25 mm × 25 mm) supplied with Milli-Q water to prevent drying damage (Fig. 2A). The gemmae on the filter paper were placed on top of an inverted 60 mm Petri dish (Fig. 2B), and the filter paper was set under the center of SDBD plasma device (Fig. 2C,D). The gemmae of *M. polymorpha* were treated for 10 s at a distance of 3 mm below the SDBD with air (temperature 22 °C and 45% humidity). The pH value of the Milli-Q water in filter paper after the SDBD plasma irradiation, and the discharge power consumed by SDBD were shown in Fig. 2E and Fig. 2F, respectively.

### Effect of the SDBD irradiation on the growth of gemmalings

The mean ± SD size and fresh weight of gemmae of *M. polymorpha* isolated from gemma cup (Fig. 1A) were 0.24 ± 0.04 mm^2^ and 14.3 ± 4.79 µg, respectively (Fig. S1). After SDBD plasma irradiation, gemmae were cultured for 10 d on 1/8 Gamborg’s B5 medium supplemented 0.6% agar (pH 5.5, adjusted by KOH) under continuous white light (approximately 40 µmol photons m^-2^ s^−1^) at temperature 22 °C and 45% humidity. MES buffer was not supplemented in this medium because it might influence growth of plants^30^.

**Figure 3:**
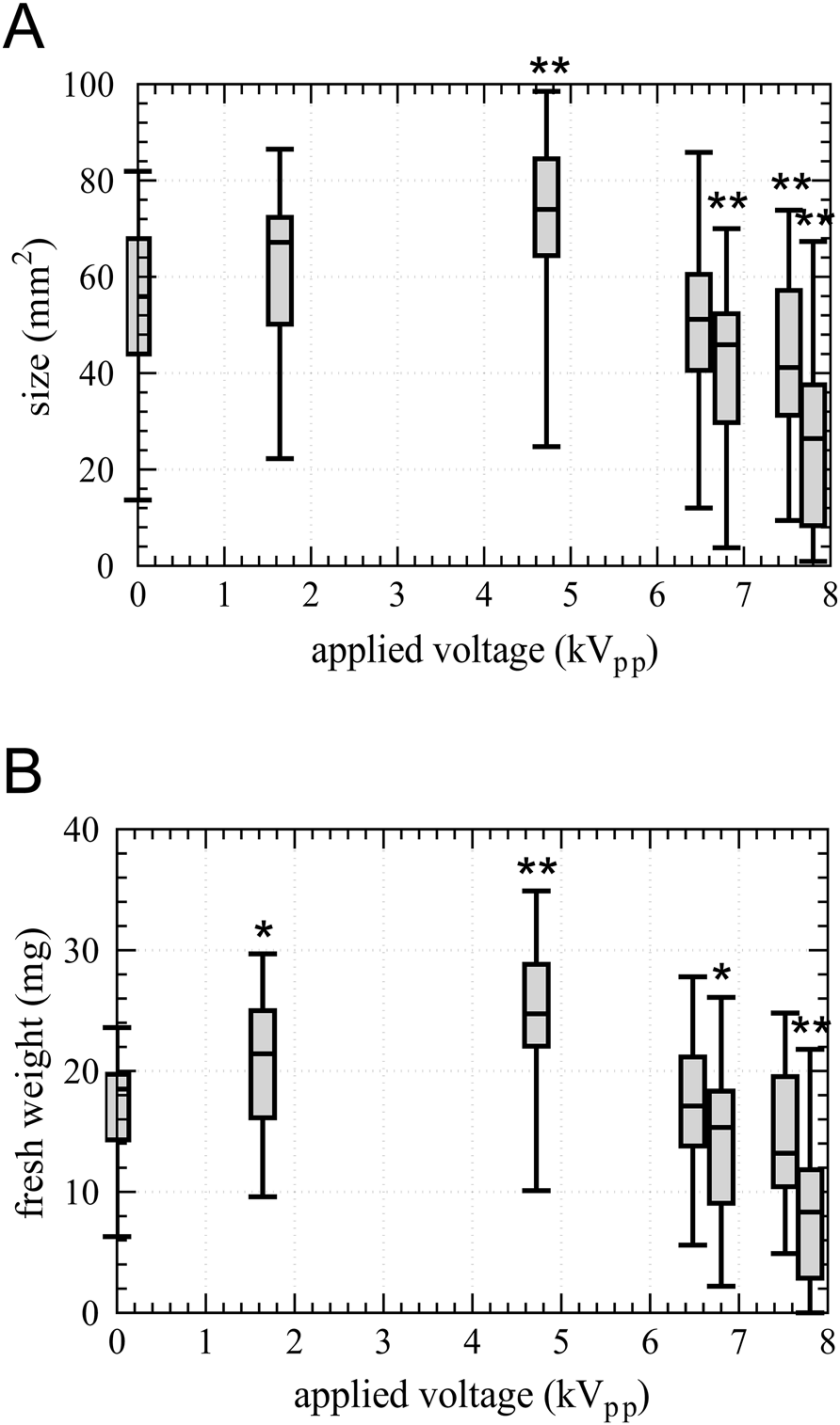
Size and fresh weight of gemmalings of *Marchantia polymorpha*. (**A**,**B**) 10 days cultured plasma irradiated gemmalings on 1/8 Gamborg’s B5 medium supplemented 0.6% agar (pH 5.5) were measured. The graphs employ box plots, illustrating minimum, lower quartile, median, upper quartile, and maximum values. A significant difference comparing to applied voltage 0 kV_pp_ as control was tested using the Brunner-Munzel test with *p < 0.05, **p < 0.01. (**A**) Size (mm^2^) of SDBD plasma treated gemmalings. (**B**) Fresh weight (mg) of SDBD plasma treated gemmalings.

Effects of SDBD plasma irradiation at various applied voltage on the mean size (mm^2^) and fresh weight (mg) of gemmalings cultured for 10 d were shown in Fig. 3A and B, respectively. Both the size and fresh weight of gemmalings increased by SDBD treatment up to discharge power of 0.47 ± 0.01 W (applied voltage of 4.72 kV_pp_) in a dose-dependent manner (Fig. 3A,B). Further increase in discharge power resulted in the decreased size and fresh weight of gemmalings (Fig. 3A,B). These results suggest that SDBD plasma irradiation at low power promotes the growth of gemmae/gemmalings, while a high treatment dose leads to growth inhibition.

### Influence of SDBD plasma irradiation on gemmalings morphology

**Figure 4:**
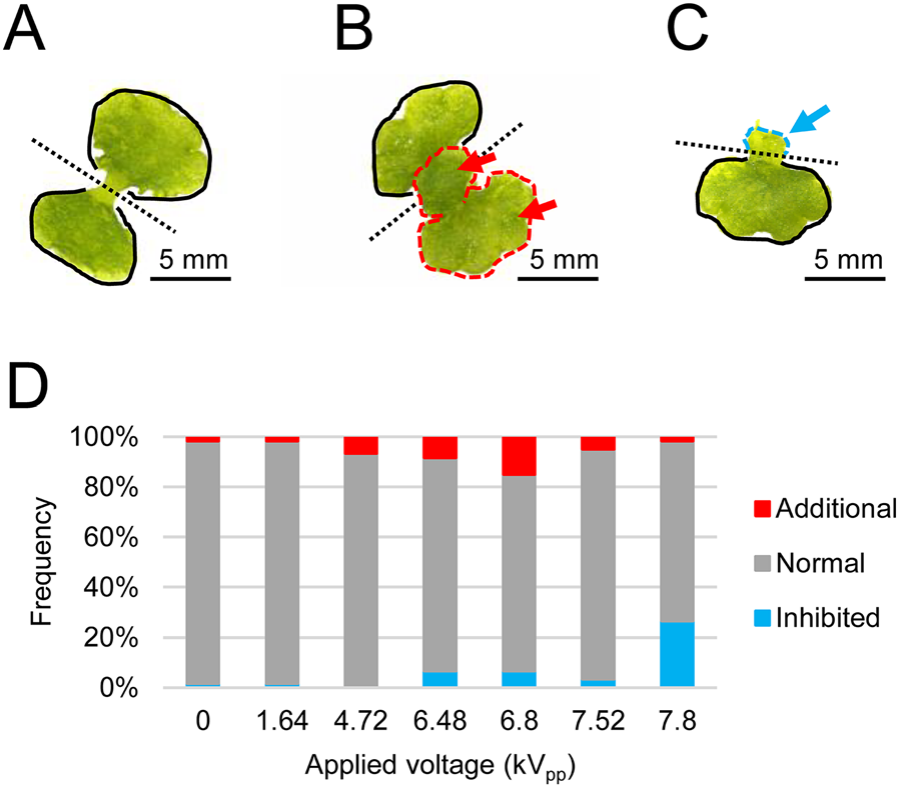
The shapes of lobes of plasma irradiated gemmalings. The representative pictures of various shapes of gemmalings in (**A**–**C**). The pictures were taken by a S8AP0 microscope (Leica Microsystems, Wetzlar, Germany) and a WRAYCAM-NOA2000 digital camera (WRAYMER, Osaka, Japan). The black dotted line in the center of gemmaling indicates the boundary between the two lobes. The black line traces the lobe of normal shape. (**A**) The normal shape of gemmaling. (**B**) The gemmaling with an additional lobe on one side lobe. Red dotted line and red arrows indicate the additional lobe. (**C**) The gemmaling with a growth-inhibited lobe. Blue dotted line and blue arrow indicates the growth-inhibited lobe. (**D**) The frequency of various shape of each lobe.

Without plasma irradiation, gemmae of *M. polymorpha* consistently grew in two nearly symmetrical lobes (normal shape^29^) in general, as shown in Fig. 4A. SDBD plasma irradiation induced two types of characteristic morphological changes in gemmalings. Representative pictures and frequency are shown in Fig. 4B,C and Fig. 4D, respectively. One type showed gemmaling with an additional lobe (Fig. 4B). Although such abnormal morphology is observed even in gemmalings without CAPP irradiation, its frequency (shown in red color in Fig. 4D) slightly increased by CAPP irradiation in a dose-dependent manner and became highest at an applied voltage 6.8 kV_pp_. Another type of morphological effect of plasma irradiation was growth inhibition of lobes of gemmalings (Fig. 4C). These lobes were generally small and shrunken but kept green and still alive. The frequency of this morphological change was shown in blue color in Fig. 4D. It was also dose-dependent and mostly induced at the highest irradiation (applied voltage of 7.80 kV_pp_).

Overall, plasma irradiation using SDBD induced three types of effects on gemmaling growth. Low applied voltage resulted in a dose-dependent enhancement of growth, without significant morphological changes. Around applied voltage 6.8 kV_pp_, there was a slight increase in the frequency of additional lobe development in a small proportion of gemmalings. At applied voltage of 7.80 kVpp, growth of gemmalings was inhibited. All three effects were distinct and occurred in a dose-dependent manner.

### Quantitative evaluation of particles by SDBD plasma

**Figure 5:**
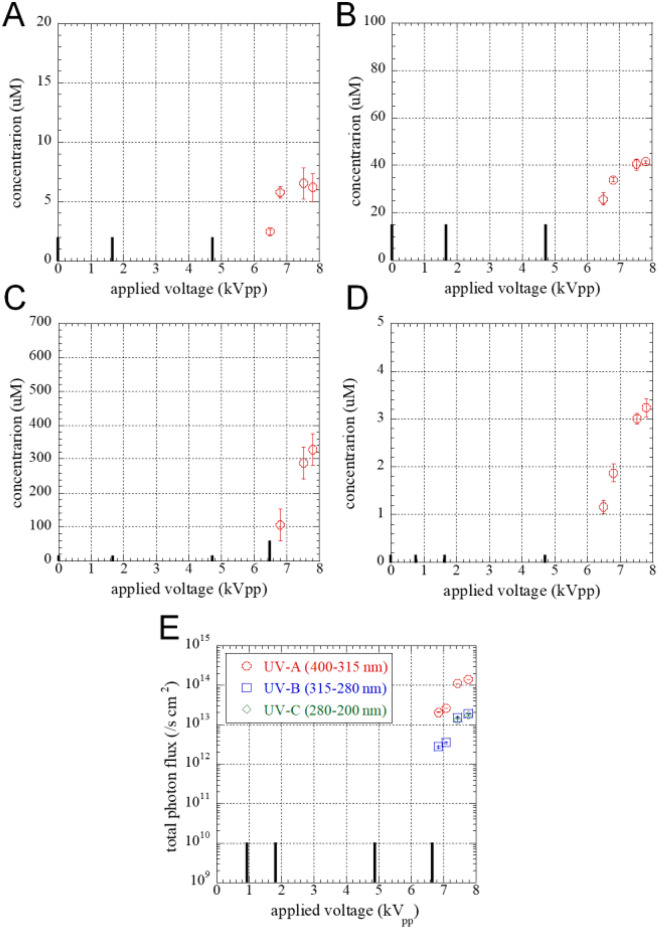
Amount of particle species delivered by SDBD plasma to gemmalings. Concentration of (**A**) H_2_O_2_, (**B**) NO_2_^-^, and (**C**) NO_3_^-^ in 240 µL of ultra-pure water in a paper filter No. 2 (25 × 25 mm) after plasma irradiation and (**D**) O_3_ in gas phase. H_2_O_2_ was measured using Hydrogen Peroxide Assay Kit ab102500 (Abcam, Cambridge, UK), NO_2_^-^ and NO_3_^-^ were measured using Griess reagent kit, NO_2_/NO_3_ Assay Kit-C II NK05 (Dojindo Laboratory, Kumamoto, Japan), and O_3_ was measured using an ozone detection tube No. 18 M (Gastec, Kanagawa, Japan). Black columns show the values under a limit of detection (LOD) at 2 µM for H_2_O_2_, 15 µM for NO_2_^-^, 15 or 60 µM for NO_3_^-^, 0.16 µM for O_3_. O_3_ concentration was obtained in ppm and converted into M using 25 °C and 1013 hPa. (E) Total photon flux of UV-A (400–315 nm) with red circles, UV-B (315–280 nm) with blue squares, and UV-C (200–280 nm) with green diamonds radiated by SDBD plasma. Photon flux was obtained by optical emission spectrum measured using a spectrometer (Avantes AVS-RACKMOUNT-EVO-USB3-5CH) with a fiber of φ1 mm, 5ch placed 3 mm away from the electrode. Black columns show the values under LOD at 10^10^ /s cm^2^.

Measuring the amount of particle delivered by SDBD plasma, such as RONS and UV photons, allows us to quantitatively discuss effects of gemmalings for plasma irradiation. Figure 5 shows that the concentrations of H_2_O_2_ (Fig. 5A), NO_2_^−^ (Fig. 5B), NO_3_^−^ (Fig. 5C) in 240 µL of ultra-pure water in a paper filter No. 2 (25 × 25 mm), and O_3_ in a gas phase (Fig. 5D) after plasma irradiation were under the limit of detection (LOD) at applied voltages below 4.72 kV_pp_. UV photon flux at 3 mm away from the electrode shows LOD at applied voltages below 6.8 kV_pp_. The concentrations of RONS and UV photon flux increased with applied voltage afterward and showed maximum values at 7.0 µM for H_2_O_2_, 41.5 µM for NO_2_^−^, 328.0 µM for NO_3_^−^, 79.1 µM for O_3_, 143.9 × 10^12^ photons s^−1^ cm^−2^ for UV-A, 19.6 × 10^12^ photons s^−1^ cm^−2^ for UV-B, and 17.7 × 10^12^ photons s^−1^ cm^−2^ for UV-C. According to the previous studies, reaction pathway of H_2_O_2_ generation is recombination of OHs, generated by H_2_O dissociation due to electron collisions^31^. There are several pathways to generate NO_2_^−^ and NO_3_^−^, triggered by vibrationally excited O_2_ and N_2_^32^. O_3_ is mainly generated by reaction of O^−^ with O_2_^33^. UV-A and UV-B originate from second positive (N_2_(C-B)) system and the first negative (N^+^_2_(B-X)) system of N_2_^34^. UV-C originates from NO γ band (NO(A–X))^35^.

## Discussion

The growth-promoting effects of CAPP irradiation have been reported in various seeds^4^. On the contrary, plasma irradiation on cotyledons and shoots of *Arabidopsis thaliana* did not display any positive effect on growth^8,9^. The gemmae of *M. polymorpha* are considered more sensitive to plasma irradiation than seeds because SDBD irradiation under the same conditions as seeds of *A. thaliana*^11^ killed most cells of gemmae of *M. polymorpha* (data not shown). In the present study, we carefully analyzed the dose-dependent effects of SDBD plasma irradiation on the growth of gemmalings of *M. polymorpha*. The dose of plasma irradiation^36,37^ depends on the applied voltages to SDBD device because the SDBD irradiation to gemmae had a fixed irradiation time (10 s) and distance (3 mm). At high applied voltage plasma irradiation, growth was significantly inhibited (Fig. 3). In contrast, plasma irradiation for voltages lower that 6.48 kV_pp_ significantly promoted the growth of gemmalings in a dose-dependent manner (Fig. 3). These results clearly indicate that CAPP irradiation has both positive and negative effects. The detail investigation of the effect less than applied voltage of 6.48 kV_pp_ and treatment time is future issues.

Growth of gemmalings was promoted at low applied voltage plasma irradiation (applied voltages below 4.72 kV_pp_), however, the pH of Milli-Q water in filter paper was not changed by low applied voltage plasma irradiation (Fig. 2E). On the other hand, the pH of Milli-Q water decreased by high applied voltage irradiation. The growth inhibition of gemmalings at high applied voltage irradiation might be affected by low pH due to the accumulation of nitric acid^38^ (Fig. 2E).

RONS, including H_2_O_2_ and nitric oxide (NO) act as signal molecules in plants^6,39^, and are crucial for the elongation of tip-growing cells such as root hairs^40,41^ and pollen tubes^42–44^ as well as lateral root emergence^45^, periclinal division during root ground tissue^46^ and cell fate at the shoot apical meristem^47^. RONS form a complex signal network among other signaling molecules (e.g., hormones, Ca^2+^, other reactive species, etc.)^39,48–50^. For instance, reactive carbonyl species (RCS), which serves as damage/signal mediators downstream of ROS, possess a chemical property enabling them to covalently modify proteins, thereby mediating ROS signals to proteins in various physiological scenarios^51^. Therefore, controlling RONS concentration transported to plants helps in regulating various events in plants. The growth of gemmalings was enhanced by irradiating the SDBD plasma at applied voltages 4.72 kV_pp_. At this treatment condition, the long-lived RONS like H_2_O_2,_ NO_2_^−^, NO_3_^−^, and O_3_ concentration were below the detection limit, as shown in Fig. 5A–D. Other short-lived species (OH, NO, etc.) were also produced in the gas phase^52^.The amount of RONS transported to the plant tissue was ideal at applied voltages 4.72 kV_pp_, the transported to promote gemmalings growth. The cause of growth promotion is still unknown, but RONS, an important signal for cell division and other processes, is the most likely candidate factor. In contrast, the growth inhibitory effect at higher applied voltage SDBD irradiation (> applied voltages 4.72 kV_pp_) caused damage by the excess RONS, as shown in Fig. 5A–D. It was reported that too much plasma-generated RONS exposure induced damage to plant^9^. Thus, RONS produced from the SDBD plasma device play an essential role in gemmalings growth promotion and inhibition. To analyze the effectiveness by RONS, further investigation of the treatment of a single active species is needed.

SDBD plasma generates UV-A (400–315 nm), UV-B (315–280 nm), and UV-C (200–280 nm), along with RONS (Fig. 5E). UV, especially UV-B, has harmful effects on organisms, such as DNA damage and oxidative damage. Our study at applied voltage 4.72 kV_pp_ showed a significant increase in size and fresh weight of gemmalings, although, at this condition, the photon flux of UV-A, -B, and -C was below the detection limit (Fig. 5E). Whereas, at a high applied voltage (> 4.72 kV_pp_), the UV photon flux of UV-A, -B, and -C increases, which results in a decrease in gemmalings size and weight. At high applied voltage (above 6.8 kV_pp_), we observed lobes with abnormal shape or damaged lobes that did not grow as shown in Fig. 4. This phenomenon is likely due to the damaging effects of RONS produced by plasma irradiation at higher applied voltages, as shown in Fig. 5. The UV flux (UV-A, -B, and -C) may also contribute to the damaging effect, whereas such damage cannot be explained by the effect of UV irradiation alone. The maximum photon density of plasma irradiation 143.9 × 10^15^ cm^−2^ for UV-A, 19.6 × 10^14^ cm^−2^ for UV-B, and 17.7 × 10^14^ cm^−2^ for UV-C is extremely lower than that, has reported to damage *M. polymorpha*, 5.11 × 10^7^ cm^−2^ for UV-A and 1.03 × 10^9^ cm^−2^ for UV-B (no data for UV-C)^53^. ​Despite irradiating the gemmalings with SDBD plasma at above 6.8 kV_pp_, a mixture of normal lobes, abnormal lobes, and inhibited growth was observed. The shape of each gemmaling may be due to its position or susceptibility during plasma treatment.

The results of this study suggest that the growth of gemmae of *M. polymorpha* can be controlled by changes in the intensity of plasma irradiation through controlling applied voltage. This makes it a suitable model system for establishing the basic concept of controlling plant growth through CAPP irradiation. Currently, the molecular mechanism behind the growth-promoting and growth-inhibiting effects of gemmalings is unknown. Investigating whether the growth-promoting effect is on either cell division or cell elongation, as well as identifying essential plasma components (e.g., RONS, UV) and plant factors (e.g., genes) involved in growth promotion and inhibition, are areas for future research.

## Conclusion

This paper proposes that gemmae of *M. polymorpha* are useful as a model plant material to study plasma agriculture. The change in power results in variation in RONS and UV concentration, affecting the gemmalings size and weight. At limited power (0.47 W) irradiation, low RONS and UV were generated, which helps in plant growth. On the contrary, high power (> 0.47 W) negatively affects gemmalings due to high RONS and/or UV values. The control environment is explicitly needed to be noticed the contribution of RONS and UV on the molecular level of *M. polymorpha*. The present experimental system using gemmalings of *M. polymorpha* would be suitable for studying the detail of the various effects of the plasma-generated RONS at the molecular level.

## Materials and methods

### SDBD setup and discharge power measurement

In research on plasma irradiation of living organisms, in addition to RONS, the effect of electric fields is often discussed in the system where the target and plasma are in contact^54^. In this study we used SDBD plasma, which can prevent plasma contact with the sample^12,13^. SDBD electrode consisted of 10 high voltage rod electrodes and 10 grounded rod electrodes. These were covered with ceramic tubes and alternately and horizontally arranged at 0.2 mm intervals so that the streamer discharge, accompanying high electric field, terminates at the ceramic surface of the grounded electrode and thus almost never reaches the sample.

Plasma was generated in air using voltage of 0–7.80 kV_pp_, 15 kHz of frequency for 10 s at temperature 22 °C at humidity 45% relative humidity (RH). SDBD electrode was placed at 3 mm away from the sample. The characteristics of SDBD plasma are shown in our previous studies^12,13^. The discharge power (Fig. 2F) *P* [W] was obtained by the V-Q Lissajous method by Eq. (1), using a capacitor of 100 nF inserted between the SDBD electrode and the ground.

1 $$P=S\times f $$

where *S* is the area of V-Q Lissajous curve [J], *f* is frequency [Hz].

### Plant materials and growth conditions

The intact gemmae from the thalli of wild type male accession line Takaragaike-1 (Tak-1), which was kindly provided by Dr. Takayuki Kohchi (Kyoto University), was used for SDBD plasma irradiation. Thalli of *M. polymorpha* were maintained under continuous white light (approximately 40 µmol m^−2^ s^−1^) at temperature 22 °C and humidity 45% on half-strength Gamborg’s B5 solid medium supplemented with 1% sucrose, 1% agar and 1 g/L MES (pH 5.5, adjusted by KOH). The gemmae obtained from 4 to 8 weeks old thalli were used for examinations. The gemmae were placed upon a paper filter with approximately 240 µL of ultra-pure water-absorbed paper filter No. 2 (Advantec, Tokyo, Japan; cut into approx. 25 × 25 mm) below SDBD electrode during the plasma irradiation. The uniformity of plasma irradiation was preliminarily validated by measuring the distribution of HNO_3_ flux under the discharge area 40 × 40 mm of SDBD electrode. The area of 25 × 25 mm at the center of the electrode gave the flux of the same order of magnitude and thus this area was adopted for uniform plasma irradiation to the sample. After SDBD plasma irradiation, gemmae were cultured for 10 days on 1/8 Gamborg’s B5 medium supplemented 0.6% agar (pH 5.5, adjusted by KOH). To avoid influence MES buffer on gemmae/gemmalings^30^, MES buffer was not supplemented in this medium. The gemmae/gemmalings were maintained under continuous white light (approximately 40 µmol m^−2^ s^−1^) at temperature 22 °C and humidity 45% RH.

Prof. Kanji Ohyama and his colleagues collected the liverwort plant named Takaragaike-1 (Tak-1) in Kyoto, and Prof. Masaki Shimamura undertook the formal identification of Tak-1 as *Marchantia polymorpha* subsp. *ruderalis*. A voucher specimen of this material has not yet been deposited in a publicly available herbarium. We have permission to collect the plant, *M. polymorpha* thalli of wild type male accession line Takaragaike-1 (Tak-1), used in this study. All plant experiments described in this manuscript were performed in accordance to relevant institutional, national, and international guidelines and legislation.

### Measurement of size and fresh weight of gemmae/gemmalings

For size measurements, images of gemmae were captured using a microscope S8AP0 (Leica Microsystems, Wetzlar, Germany) paired with a digital camera WRAYCAM-NOA2000 (WRAYMER, Osaka, Japan). Gemmalings were photographed using a digital camera EOS M5 (Canon, Tokyo, Japan). The fresh weight measurements were conducted using an electronic balance HR-202i (A&D, Tokyo, Japan). In the case of 10-day cultured gemmalings, we meticulously extracted them from the solid medium, ensuring the removal of excess water by thorough absorption with filter paper.

### Data analysis of size and fresh weight of gemmae/gemmalings

The size of gemmae/gemmalings was determined utilizing ImageJ/Fiji software, based on the pictures. Graphs illustrating the size and fresh weight of gemmae/gemmalings (Fig. 3A,B, Fig. S1A,B) were generated using GraphPad Prism software. Statistical analyses were performed using R software.

### Measurement of H_2_O_2_, NO_2_^−^, NO_3_^−^ and O_3_ concentration

The concentration of hydrogen peroxide H_2_O_2_ in 240 µL of ultra-pure water in a paper filter No. 2 (25 × 25 mm) were measured after plasma irradiation for 10 s by Hydrogen Peroxide Assay Kit ab102500 (Abcam, Cambridge, UK) and a micro plate reader SynergyHT (Biotec, Tokyo, Japan). The measurement was performed based on the product protocol. Sample solution was collected in a quartz Petri dish and used for measurement. LOD of H_2_O_2_ was determined as 2 µM based on the standard curve (Fig. S2). The wavelength of the micro plate reader was set as 570 nm. The concentrations of nitrite ion NO_2_^−^ and nitrate ion NO_3_^−^ in 240 µL of ultra-pure water in the paper filter were measured using NO_2_/NO_3_ Assay Kit-C II NK05 (Dojindo Laboratory, Kumamoto, Japan) and the micro plate reader. The sample solution was collected and diluted to secure the required amount 160 µL and more diluted as the measured values exceeded the measurable range 10–100 µM based on standard curves (Fig. S3). According to the protocol, LOD for NO_2_^−^ and NO_3_^−^ was 10 µM. The dilution factor and practical LOD was 1.5-fold (15 µM) for NO_2_^−^, 1.5-fold (15 µM) for NO_3_^−^ as the applied voltage ≤ 4.72 kV_pp_, and sixfold (60 µM) for NO_3_^−^ as the applied voltage > 4.72 kV_pp_. The obtained values were corrected as the concentrations by multiplication by corresponding dilution factors. The wavelength of the micro plate reader was set as 540 nm. The concentration of O_3_ in a gas phase was measured immediately after plasma irradiation using an ozone detection tube No. 18 M (Gastec, Kanagawa, Japan) through a hole of glass plate vertically placed at 3 mm away from the center of the SDBD electrode^55^. The hole was connected to the gas test tube through a silicone rubber tube. The gas path from the hole and the ozone detection tube was a cylinder shape with 200 mm of the length and φ5 mm of the radius. The measured value was corrected by the dead volume of the gas path calculated as 3.927 mL. LOD is 4 ppm (= 0.16 µM) as T_g_ = 25 °C and 1013 hPa, molecular weight 48 g/mol.

### Measurement of UV photon flux

The UV photon flux was obtained using optical emission spectrum (OES). OES was measured under dark by a fiber (φ1 mm, 5 channels) and an absolutely calibrated spectrometer AVS-RACKMOUNT-EVO-USB3-5CH (Avantes, Apeldoorn, Netherlands) with 10 s of integration time. The photon flux $\Gamma$ [/s cm^2^] was calculated by Eq. (2).

2 $$\Gamma =\frac{I \times \delta }{E}$$

where *I* is a spectral irradiance [J/s cm^2^/nm], $\delta$ is a wavelength interval [nm], and *E* is an energy [J] of a photon with a wavelength λ [m], derived by Eq. (3).

3 $$E=h\nu =h\frac{c}{\lambda }$$

where *h* is Planck constant as 6.626 × 10^–34^ [J s], *c* is the speed of light, and *v* is frequency [Hz]. The signal was 10^10^ /s cm^2^ as no voltage was applied, thus this value was defined as the limit of detection (LOD).

### Supplementary Information

Supplementary Figures.

## Data Availability

Raw data will be provided upon request.

## Abbreviations

CAPP: Cold atmospheric pressure plasma
RONS: Reactive oxygen and nitrogen species
SDBD: Scalable dielectric barrier discharge
Tak-1: Takaragaike-1
