# Supplementary material for: Growth control of Marchantia polymorpha gemmae using nonthermal plasma irradiation
Source: Sci Rep. 2024 Feb 7;14:3172. doi: 10.1038/s41598-024-53104-1 (PMC10850213; doi:10.1038/s41598-024-53104-1)
Supplement: Supplementary file 1 — Supplementary Figures. [file 41598_2024_53104_MOESM1_ESM.docx]

**Supporting information**

**Growth control of *Marchantia polymorpha* gemmae using nonthermal plasma irradiation**

**Shoko Tsuboyama^1^, Takamasa Okumura^2^, Pankaj Attri^3^, Kazunori Koga^2^*****, Masaharu Shiratani^2, 3^, and Kazuyuki Kuchitsu^1^****

^1^Department of Applied Biological Science, Tokyo University of Science, 2641 Yamazaki, Noda city, Chiba, 278-8510, Japan

^2^Faculty of Information Science and Electrical Engineering, Kyushu University, Fukuoka 819-0395, Japan

^3^Center of Plasma Nano-interface Engineering, Kyushu University, Fukuoka 819-0395, Japan

These authors contributed equally: Shoko Tsuboyama and Takamasa Okumura

**Co-corresponding Authors:**

*Kazunori Koga

744 Motoka, Fukuoka city, Fukuoka, 819-0395, Japan

Tel: +81-92-802-3716

Fax: +81-92-802-3717

E-mail: koga@ed.kyushu-u.ac.jp

**Kazuyuki Kuchitsu

2641 Yamazaki, Noda city, Chiba, 278-8510, Japan

Tel: +81-4-7122-9404

Fax: +81-4-7123-9767

E-mail: kuchitsu@rs.tus.ac.jp

Figure captions

**Fig. S1** Size and fresh weight of non-cultured gemmae of *M. polymorpha*.

**Fig. S2** Standard curve for H_2_O_2_ concentration.

**Fig. S3** Standard curve for concentration of nitrite ion NO_2_^-^ and nitrite ion NO_2_^-^ and nitrate ion NO_3_^-^.


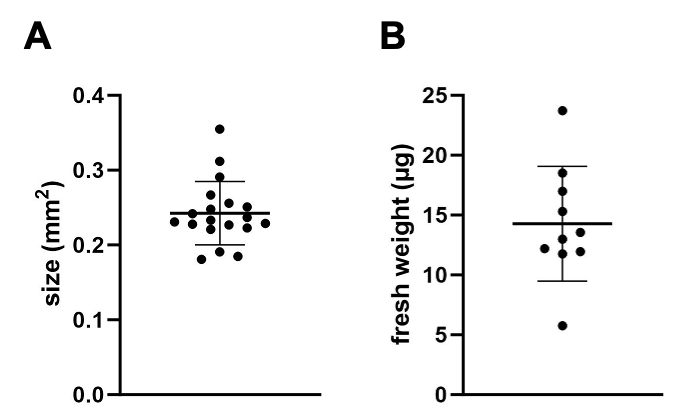


**Fig. S1**

Size (A) and fresh weight (B) of non-cultured gemmae of *Marchantia polymorpha*. Each dot shows raw data. The bar and the error bar represent the mean value and the standard deviation, respectively.

**Fig. S2**

Standard curve for H_2_O_2_ concentration. Standard curve was obtained by colorimetry using Hydrogen Peroxide Assay Kit ab102500 (Abcam, Cambridge, UK) and a micro plate reader SynergyHT (Biotec, Tokyo, Japan). The OD value with the concentration was obtained at absorption at 570 nm (standard curve equation: y = 0.0138x - 0.0284, R² = 0.997). The measurement was performed according to the protocol.

**B**

**A**


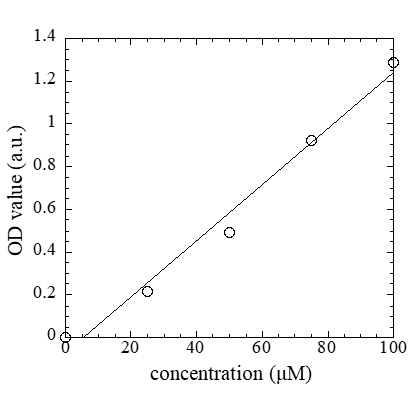

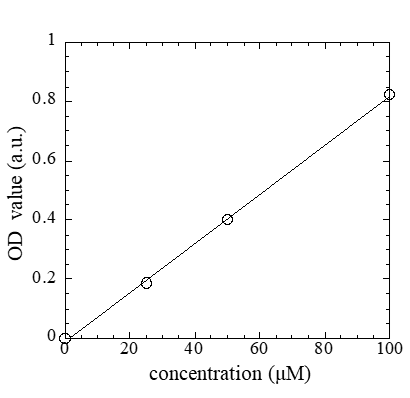


**Fig. S3**

Standard curve for concentration of (A) nitrite ion NO_2_^-^ and (B) nitrite ion NO_2_^-^ and nitrate ion NO_3_^-^. Standard curves were obtained by colorimetry using NO_2_/NO_3_ Assay Kit-C II NK05 (Dojindo Laboratory, Kumamoto, Japan) and a micro plate reader SynergyHT (Biotec, Tokyo. Japan). Based on the product protocol, the concentration of NO_3_^-^ was obtained by the equation; [NO_3_^-^] = [NO_2_^-^ + NO_3_^-^] – [NO_2_^-^]. The OD values with the concentration were obtained at absorption at 540 nm. The standard curve specifications were y = 0.0131 x - 0.0712 (R^2^ = 0.984) for NO_2_^-^ and y = 0.00828 x – 0.0102 (R^2^ = 0.999) for NO_2_^-^ and NO_3_^-^. The measurement was performed according to the protocol.
